# Dr. Paterson, on Vaccine Inoculation

**Published:** 1801-07

**Authors:** D. Paterson

**Affiliations:** Montrose


### *To the Editors of the Medical and Phyfical Journal.*

GENTLEMEN,

YOU may, *if you fhall deem it proper*, give the inclofed a place in your Journal.

I have the honour to be, &c.

*Montrofe, 25th May,* 1801. D. PATERSON.

---

WHEN we contemplate the Vaccine Inoculation, we are at once loft in admiration and gratitude; and while we are irrefiftibly led to join the wondering, the grateful throng, in paying the juft tribute of applaufe to Dr. Jenner, the immortal difcoverer, we muft, at the fame time, confefs how much we are indebted to the ingenious and benevolent Dr. Pearfon for bringing, in fuch a handfome manner as he did, the bufinefs before before the public, thereby exciting, all at once, an univerfal, an unparalleled qneft of inveftigation, and furnifhing, by innumerable and fatisfactory experiments, a complete confirmation of the noble difcovery. Nor will candour allow us to forget, that, as a valuable medium of information on this important fubject, your no lefs widely circulated than ufeful Journal has held a very diftinguifhed rank.

With refpect to the Vaccine Inoculation, were individual teftimony neceffary, mine, according to the experience I have, fhould not be wanting: But, in truth, after the very honourable teftimonial that appeared in the 18th Number of your Journal, bearing the names of almoft all the eminent medical practitioners in London, for the very exprefs purpofe of confirming all that had previoufly been advanced in favour of the Vaccina, by the Difcoverer, by Dr. Pearfon, by Dr. Woodville, and by all thofe gentlemen whofe names had appeared in your Journal to detailed cafes of the difeafe: I fay, after fuch an immenfe body of evidence, can the mere name of any individual, whether he refides near, or is far removed from the metropolis, ferve any other purpofe befides that of fhewing, if not worfe, the vanity of the writer?

No lefs for the fake of medicine in general, than for Vaccine Inoculation in particular, I was forry, nay, extremely forry to obferve in the 20th Number, page 340, of the Medical and Phyfical Journal, a letter, in all the extravagance of pompofity, dated Montrofe, 10th September 1800, from Dr. A. Wilfon, giving his authority in favour of the new practice.

Dr. W. in his letter, fets off by faying, "If the information of an individual practitioner, far removed from the metropolis, can add any thing to the very high medical authority with which you have favoured the public on the fubject of Vaccine Inoculation, my teftimony is very much at your fervice; it may in fome meafure, ferve to afcertain the progrefs of that very ufeful difcovery all over this ifland." Then the Dr. after pompoufly ftating, that, "in the month of July laft" the Small-pox appearing in the neighbourhood of fome of the firft families, he propofed Vaccine Inoculation, fays, in the conclufion of his letter: "My propofal was readily agreed to; Vaccine matter was immediately procured; the children were inoculated, and took the infection in the moft diftinct and favourable manner; the fears of the parents were completely removed; and from this fuccefs, and the examples of the firft families, I am happy to inform you, that Vaccine Inoculation is becoming general in this diftrict of country."

Now, Gentlemen, with regard to the vaft information, the great teftimony of Dr. Wilfon; with refpect to the children taking taking the infection in the moft diftinct and favourable manner; and alfo, with refpect to the fears of the parents being completely removed, the truth is, that when the Dr. wrote that very extraordinary letter, he had not inoculated above eight or nine children (for I would not by any means fall fhort of the mark) with Vaccine matter; and what ferves to render the bufinefs the more ridiculous, is, that not only fome of thofe very few cafes had, at that time, after repeated attempts, proved unfuccefsful, but even none of the fuccefsful ones had been tried with variolous matter. And as to Dr. Wilfon's fuccefs being the means of rendering the Vaccine Inoculation general in this diftrict, I am under the difagreeable neceffity of obferving, that the Dr. had no more right to claim any merit from the latter than he had caufe to boaft of the former: For, it muft be confeffed, that after very long and very great exertion on the part of moft of the other medical practitioners, whofe attention, totally independent of Dr. Wilfon, was very early and very fuitably called to this no lefs novel than interefting bufinefs, by the communications of Drs. Jenner and Pearfon, the new practice is, even now, by no means general in this place; and no wonder, when our well-meant endeavours, inftead of being aided, are thwarted by fuch information and fuch teftimony as have been quoted. I wifh not, however, to offer any further comment on the letter in queftion.

Thus far, Gentlemen, I have, I prefume, been in the way of my duty. Now, purfuing the fame path, but varying the object a little, I muft beg leave to trouble you with a very concife, though accurate hiftory of my proceedings in the bufinefs of Vaccine Inoculation, and alfo with fuch remarks, in a plain way, as my practice and that of moft of my fellow practitioners has afforded, and as may, I humbly think, have fome fmall tendency to promote the general good.

In the year 1799, thinking well of the Vaccine Inoculation, from the highly refpectable teftimony of Dr. Jenner and Dr. Pearfon, I began to prepare the minds of people here for its reception; and by the following fummer, I was happy to find I had fucceeded in making a favourable impreffion on many. But candour obliges me to fay, that the greater part of my medical brethren here, were alfo employed in a fimilar manner.

In June 1800, I requefted, and immediately obtained, a fupply of Vaccine matter from Dr. Thomas Anderfon of Leith, who, as far as I have heard, ftands firft on the lift of Vaccine Inoculators in this divifion of the kingdom, and who, with a degree of promptnefs and liberality which does him no inconfiderable honour, has fent fupplies of matter to a great number of medical practitioners in different parts of the country.

Dr.

Dr. Anderſon, I underſtand, got his original ſupply of matter from Dr. Jenner.

With the matter I obtained from Dr. Anderſon, I began to practiſe Vaccine Inoculation in this town, making a tender of my ſervices to the lower claſs of people gratis. My firſt attempts, however, were not ſo ſucceſsful as I could have wiſhed. Indeed, from the very high temperature of the air, and from the pertuſſis becoming more and more general, I gave up the buſineſs for a while; but when the weather became cool, and pertuſſis leſs prevalent, I again obtained matter from Doctor Anderſon, by thread, with which I inoculated four children, three of whom went through the diſeaſe. From thoſe caſes I have been going on ſlowly, but conſtantly, ever ſince; and, from that ſource, I have had the ſatisfaction of giving ſupplies of matter to almoſt all the medical practitioners here, as well as to ſome in the neighbourhood, including a lady, who, from motives of humanity, has become a keen inoculator in her vicinity. Dr. Hunter, Dr. Gibſon, Mr. Crabb, Mr. Alexander, practitioners here, and myſelf, for the purpoſe of multiplying our opportunities of making obſervations, and of extending our knowledge, have been in the habit of viſiting one another's patients, and of freely communicating to each other our ſentiments reſpecting the new and important practice. We have inſerted the matter in all the various ways, both as to method and time; and alſo, we have, ſubſequent to the Vaccine, ſubjected a variety of our patients to the Variolous Inoculation. Since we commenced the new practice, to the preſent time, we find, by our liſts, we have inoculated 321 perſons, from the age of five weeks to twenty-one years, excluſive of 44 inoculated by the lady alluded to above, making altogether 365; and, from all that has been obſerved, I have been led to make the following remarks.

1. That the genuine, or, as we may term it, the ſtandard Vaccina, is a mild diſeaſe, and may, not only with ſafety but advantage, be communicated to children at any period during teething, or when their conſtitutions have been weakened, or rendered irritable, by teething, as well as when they labour under many other complaints to which they are ſubject, providing there be no fever, or any ſerious eruption preſent. Many children, puny from teething, one with diarrhœa, and another with a ſlight eruption and cough, from the ſame cauſe, but al· without fever, as well as one child labouring under pertuſſis ſine pyrexia, have been obſerved to get very faſt rid of the complaints under which they laboured, and improve much in their general health, after the Vaccina.

2. That, although it has hitherto been obſerved by many that the Vaccina has ſhewn itſelf ſubject to variations, both with with refpect to time and appearance, yet, from all that I have feen, it may, in a very general fenfe, be confidered as regular and uniform in its fymptoms and progrefs.

3. That the Vaccina has come to its acmé fometime between the 8th and 14th day; very generally on the 9th or 10th, fometimes on the 11th, but rarely, I prefume, on the 12th, and more rarely on the 13th or 14th day. In 365, inoculated as above, only two 12th day cafes, and one 14th day cafe have occurred here; but no 13th day cafe has been feen.

4. That hitherto, however, fome of the irregularities alluded to in the fecond remark, as well as others, have not, it is probable, proceeded fo much from any thing peculiar in the nature of the genuine Vaccina, or in the human conftitution, as from inexperience, in not having known how to render the general phenomena of the difeafe more regular and uniform.

5. That the genuine or ftandard Vaccina, by rendering the human conftitution unfufceptible of the action of Variolous matter, has proved, in a variety of inftances highly fatisfactory, a preventative of Small-pox.

6. That often, although the local difeafe has been highly characteriftic, yet the conftitutional affection has been very flightly marked; fometimes, indeed, fo very flightly, that a whitifh tongue, at other times a fomewhat accelerated pulfe, with a hot fkin, for a very fhort while, has been the only perceptible fymptom of indifpofition.

7. That, vice verfa, as in the cafe of one of my own patients, which fhall be narrated hereafter, though the local affection may be very indiftinct, yet the conftitutional difeafe may be completely and ftrongly marked, even to three or four hours of delirium. This however will, it may be prefumed, very rarely occur; for, in 365 cafes, it has only happened once; and, befides, in no communication hitherto have I feen it remarked.

8. That, inftead of the genuine or ftandard difeafe, anomalous, and even fpurious or inefficient cafes have occurred, moft likely, and in a very general fenfe, from the matter having been taken when the difeafe has been far advanced, or from its having been kept too long on lancets, glafs, or thread, thereby lofing its purity, and its fpecific energy.

9. That other difeafes may interfere with the Vaccina, and from their anomalous appearance may, if not ultimately deceive, for fome little time puzzle the inexperienced practitioner, as will be exemplified by a cafe.

10. That although fome anomalous cafes, I mean fome of thofe running into broad, high, darkifh coloured crufts or fcales, but at the fame time accompanied with fomething very like the fpecific inflammation, and attended more or lefs with febrile fymptoms,

ſymptoms, as mentioned by Dr. Pearſon, have, in the end, proved very ſatisfactory; yet, in my humble opinion, all anomalous caſes, not only from their being ſuſceptible of great variety, but as it may, at leaſt for a time, be exceedingly difficult, nay impoſſible, to diſtinguiſh the efficient from the inefficient ones, ought to be conſidered as ſuſpicious.

11. That all caſes ought to be looked upon as doubtful when the inoculated part does not inflame on the third, fourth, fifth, or ſome ſubſequent day, and become a bluiſh white, or whitiſh veſicle, containing a limpid fluid, moderately increaſing in ſize, dimpling in the middle, with ſurrounding inflammation, which, in a very general ſenſe, increaſes after the ſeventh or eighth day, followed very commonly on the ninth or tenth day by febrile ſymptoms more or leſs perceptible, and leaving very frequently as it goes off, a haló-like circle, or ſometimes even two ſuch circles, round the veſicle, which by this time is degenerating, from its centre to its circumference, into a darkiſh coloured and moſt commonly dry cruſt, very ſimilar to the delineation of Mr. Walker, in the caſe of his own child. (Vide Med. and Phys. Journal, Vol. I.)

12. That, the more certainly to enſure ſucceſs in Vaccine Inoculation, I mean to produce the genuine Vaccina, and that very generally regular and uniform in its phenomena, the fluid to inoculate with ought to be taken directly from one perſon to another, on the 6th, 7th, or 8th day, or, what perhaps would be a better rule, while it is limpid, any time after it is formed in the veſicle, and before the inflammation is much increaſed; becauſe when the inflammation round the veſicle has increaſed much (and ſometimes it ſpreads very rapidly) there is either no fluid in the veſicle, or what it contains, inſtead of being limpid as it ought to be, is of a purulent-like appearance, and may fail in producing the deſired effect—the genuine or ſtandard diſeaſe.

13. That, with regard to the inſertion of the matter, inciſion or puncture will, if properly made, anſwer the purpoſe equally well. But one-eighth part of an inch, or a very little more, is a ſufficient length for the inciſion; and neither it nor the puncture ought to be deeper than the cuticle.

14. That if, contrary to deſign, the wound ſhould happen to be made deeper than the cuticle, and ſome drops of blood iſſue from it, the Vaccine matter ought not to be inſerted until the bleeding be ſtopped, otherwiſe the matter will run a great riſk of being waſhed away, or of forming, with the blood, a dry inert cruſt, and, conſequently, the operation of failing in its wiſhed for reſult.

15. That if dry Vaccine matter, preſerved on glaſs, is to be

 uſed, ufed, the fame method as has already been mentioned may be purfued; but the matter ought firft to be rendered fluid by means of a very fmall quantity of cold water.

16. That if a thread, impregnated with Vaccine matter, and dry, is to be ufed, a fmall puncture, as already recommended, with a very narrow fpear-pointed lancet, ought to be made, and a little bit of the impregnated thread, previoufly moiftened as above, may be introduced into it by means of the fharp end of a fmall probe, and there allowed to remain.

17. That gently fcratching acrofs the arm with the point of a lancet, armed with frefh wet Vaccine matter, taken at a proper time, until the cuticle be juft divided, has, of all other methods, anfwered beft. For inftance, I take hold firmly of the patient's arm at the elbow, with my left hand, and with a lancet, armed with proper Vaccine matter, between the thumb and fore-finger of my right hand, and with its point downwards, ufing as a fulcrum my right little finger on the patient's arm, I make a fcratch between one-eighth and one-fourth of an inch long, acrofs the arm, by gentle and repeated ftrokes from fide to fide, until there is the very flighteft appearance of blood, and then wipe off on the wound any matter that remains on the fides of the lancet. During the whole of this operation the matter is defcending from the lancet into the little wound that is making. It is worthy of remark that, lately, this method has not once failed in above a hundred cafes.

18. That the Vaccina has not been obferved to fuperinduce any particular difeafe.—In fome cafes a fmall flight eruption, unattended with any the leaft diftreffing fymptom, and of fhort duration, has been feen a fhort time after to follow it. In the cafe of one child, indeed, five weeks old, a very extenfive and troublefome eruption foon fucceeded the difeafe; but whether it had the Vaccina for its caufe, would, perhaps, be a very hard matter to determine, as the child had a flight eruption fome little time before the inoculation took place; and alfo, as another child, ten weeks old, puny, with a cough and fmall eruption, went through the difeafe in a very diftinct and mild manner, and evidently with great advantage. On the circumftance of the firft mentioned child, however, the caution in the firft remark is founded.

19. That no topical applications have been found in the fmalleft degree neceffary in cafes of the genuine Vaccina, the local affection having always healed very foon, and in the kindeft manner; but not fo in fpurious cafes of the difeafe, where improper matter has been ufed!

20. That no inftance of the Vaccina occurring twice in the fame

ſame perſon has been obſerved; nor has the Vaccina ever been produced after the Small-pox.

21. That in ſome patients, flat topped puſtules with inflamed baſes, on parts of the body diſtant from the inoculated part, have been obſerved; they appeared on the third or fourth day after the febrile ſymptoms, which were very ſmart, began to ſubſide. I have inoculated with matter from ſome of thoſe puſtules, but without effect.

22. That I have run Vaccine matter, taken on the ſeventh or eighth day, but generally on the former, twenty times thro' the human conſtitution, without being able, on attentively comparing the firſt and laſt caſes of the ſeries, to note any perceptible difference in its effects.

23. That, in 365 perſons inoculated, not one death, nor, I believe I may venture to ſay, any untoward circumſtance has happened in conſequence of the Vaccine Inoculation.

I have taken the liberty of being the more particular with reſpect to the phenomena of the Vaccina, the anomalous and inefficient caſes, the time of taking the matter, and the manner of inſerting it; becauſe I have juſt heard, that, in different places, many children who had previouſly, as was ſuppoſed, gone through the Vaccine diſeaſe, have ſince been ſeized with the natural Small-pox; and further, becauſe I am very much afraid, that, throughout this kingdom, as well as on the continent, &c. more inefficient caſes than we are aware of, have already occurred. Inexperience, no doubt, has been, and will be, the cauſe of many errors; but inattention, which is leſs excuſable, has, I am apt to think, been productive of conſiderable miſchief. I have lately heard of ſome gentlemen, who from the pureſt motives inoculate paupers gratis, but who never ſee their patients after inſerting the matter. The impropriety of ſuch a plan is ſo evident, that while we applaud the intentions of ſuch gentlemen, we muſt certainly condemn their practice.

I ſhall now bring the preſent communication to a concluſion, by offering you two extraordinary caſes that have happened in my own practice, which will ſerve not only to illuſtrate ſome of the obſervations I have made, but along with others of my remarks not leſs important, to ſhow the great neceſſity of particular attention in the buſineſs of Vaccine Inoculation.

Case I. (Alluded to in the 9th remark.) On the 2d of July, 1800, I inoculated a healthy boy, thirteen months old, with Vaccine matter. On the third day from the inoculation the inciſion was inflamed, as I thought ſpecifically, and continued ſo all that and the fourth day; but on the fifth day the inflammation was almoſt gone, and by the eighth day it, as well as the inciſion, was obliterated. On the tenth day, however, the child unexpectedly laboured under ſymptoms of fever, and on the morning of the eleventh day a very ſingular eruption made its appearance, conſiſting of about ſixty puſtules, ſcattered over all the ſurface, but chiefly occupying the trunk and thighs, of a purpliſh hue, with inflamed baſes, from the ſize of a ſmall pin-head to that of a pea, and containing a limpid fluid. As the eruption advanced, the febrile ſymptoms diſappeared. The puſtules continued increaſing in number until the thirteenth day from the inoculation, amounting altogether to about ninety, dimpling in their centres as they maturated, and at laſt forming brown cruſts, which deſquamated in a few days.

This caſe, not from its ſeverity, nor from the leaſt apprehenſion of ultimate danger, but from its ſingularity in a noſological ſenſe, puzzled me. The eruption was uncommon and curious. That it was the Vaccina I was by no means ſatisfied. I invited four of my medical brethren, well informed men, to look at it, all of whom confeſſed they never had ſeen ſuch an eruption. I had an opportunity of ſhewing it alſo to a very worthy experienced medical friend, who had ſeen a great number of Dr. Woodville's patients under the Vaccina, in London, and even he declined giving a decided opinion reſpecting this anomalous caſe.

The diſeaſe, the ſymptoms of which I have here detailed, was evidently of the claſs Pyrexiæ, and, I preſume, a variety of ſome one of the ſlighter Exanthemata, perhaps of Varicella; as, according to after information, but by no means authentic, a few caſes of that malady were obſerved in this place a ſhort time before. At all events, it was ſomething remarkable that that diſorder ſhould have interfered ſo very cloſely with the inoculation. Pray, could that have prevented the latter from producing its ultimate effect? or, could this have operated in altering the ſhape of the former? At any rate, in conſequence of not being pleaſed with the reſult of this attempt, I ſubjected the child again to Vaccine Inoculation with effect.

Case II. (Alluded to in the 7th remark.) On the 17th of October, 1800, a robuſt healthy boy, ſix years old, was inoculated with Vaccine matter. 21ſt, The inoculated part ſlightly inflamed. 22d. Much the ſame as yeſterday. 23d. Inflammation ſomewhat increaſed. 24th. Much the ſame as yeſterday. 25th. Inflammation conſiderably diminiſhed. 27th. Inflammation almoſt entirely gone. About noon, this day, he began to complain of head-ach, thirſt, &c. with a ſlight degree of delirium towards evening; was in bed from dinnertime time. 28th. A very ſlight degree of redneſs in the inoculated part; pulſe 108; tongue whitiſh; but without any particular complaint. 29th. Inoculated part perfectly well; no general complaint. 31ſt. Three puſtules with flat tops and inflamed baſes, containing a limpid fluid, and, in a word, bearing all the characteriſtic marks of Vaccine puſtules, were obſerved on his upper lip and noſe. This patient, ſome time afterwards, was again inoculated with Vaccine matter, when the inoculated part inflamed on the ſecond day, and ran faſt into a ſcab, diſcharging a purulent-like matter. The ſame boy, nearly ſix months after the firſt inoculation, was carefully inoculated with Variolous matter, which had no other effect than that of producing a ſlight degree of inflammation in the inciſed part.

